# High Expression of H3K27me3 Is an Independent Predictor of Worse Outcome in Patients with Urothelial Carcinoma of Bladder Treated with Radical Cystectomy

**DOI:** 10.1155/2013/390482

**Published:** 2013-09-04

**Authors:** Jianye Liu, Yonghong Li, Yiji Liao, Shijuan Mai, Zhiling Zhang, Zhouwei Liu, Lijuan Jiang, Yixin Zeng, Fangjian Zhou, Dan Xie

**Affiliations:** ^1^State Key Laboratory of Oncology in South China, Sun Yat-sen University Cancer Center, Guangzhou, Guangdong 510060, China; ^2^Department of Urology, Sun Yat-sen University Cancer Center, Guangzhou, Guangdong 510060, China; ^3^Department of Pathology, Sun Yat-sen University Cancer Center, Guangzhou, Guangdong 510060, China

## Abstract

It has been suggested that trimethylation of lysine 27 on histone H3 (H3K27me3) is a crucial epigenetic process in tumorigenesis. However, the expression pattern of H3K27me3 and its clinicopathological/prognostic significance in urothelial carcinoma of bladder (UCB) are unclear. In this study, upregulated expression of H3K27me3 protein was observed in the majority of UCBs by Western blotting. High expression of H3K27me3 was examined by IHC in 59/126 (46.8%) of UCB tissues and in 18/72 (25.0%) of normal urothelial bladder epithelial tissues (*P* = 0.002). High expression of H3K27me3 was associated with multifocal tumors and lymph node metastases (*P* < 0.05). Patients with high expression of H3K27me3 had shorter cancer-specific survival (CSS) time than patients with low expression of H3K27me3 (*P* < 0.001). In different subsets of UCB patients, high expression of H3K27me3 was also a prognostic indicator in patients with grade 2 and grade 3, pT1, pT2, pT3, and pN− disease (*P* < 0.05). Importantly, expression of H3K27me3 was an independent predictor for CSS (*P* < 0.001) of UCB patients treated with radical cystectomy (RC). Our data suggests that high expression of H3K27me3 is an independent molecular marker for predicting poor prognosis of UCB patients treated with RC.

## 1. Introduction

Urothelial carcinoma of bladder (UCB) is one of the major causes of morbidity and mortality in Western countries [1]. Clinically, radical cystectomy (RC) remains the most common treatment for patients with muscle-invasive UCB or for patients with superficial disease that is at high risk of recurrence and progression. Despite advances in surgical technique and an improved understanding of the role of pelvic lymphadenectomy, the 5-year cancer-specific survival (CSS) remains at only 50–60% [2, 3]. In addition, while providing important prognostic information on UCB, the currently clinical and pathological variables have a limited ability to predict tumor recurrence, progression, and/or patient survival. The most possibly underlying reason might be the heterogeneous biological properties of UCB. Therefore, the search for specific genes alterations which determine biological nature and behavior of UCB would be of utmost importance to optimize individual therapy. However, such reliable biomarkers are still substantially limited. 

It has been reported that epigenetic changes are involved in the silencing of various tumor-suppressor genes, the facilitation of tumorigenesis, and/or the progression of human cancers [4–6]. Histone methylation has been found to play an important role in regulating gene expression and chromatin function [5]. Trimethylation of lysine 27 on histone H3 (H3K27me3), a transcription-suppressor histone modification, is catalyzed by enhancer of zeste homolog 2 (EZH2) [7]. EZH2, the catalytic subunit of the polycomb repressive complex 2 (PRC2), contributes to the maintenance of cell identity, cell cycle regulation, and tumorigenesis. EZH2 is frequently overexpressed and correlates with poor prognosis in many human cancers [8–12], as well as in UCB [13, 14]. Up to date, however, the protein expression of H3K27me3 in UCB and its associated clinicopathological and prognostic significance have not been investigated. Thus, in the present study, we aimed to investigate the clinical/prognostic implication of H3K27me3 in UCB patients treated with RC.

## 2. Material and Methods

### 2.1. Patient Information and Tissue Samples

In this study, for analysis of H3k27me3 protein levels in UCBs by Western blot, 15 pairs of fresh UCB and adjacent morphologically normal bladder tissues underwent RC frozen and stored in liquid nitrogen until further use. In addition, for preparation of the bladder tissue microarray (TMA), 126 patients with UCB that underwent RC were selected from the surgical pathology archives of the Department of Pathology, Cancer Center, and the First Affiliated Hospital, Sun Yat-Sen University, between 1999 and 2008. Prior patients' consent and approval from the Institutional Research Ethics Committee of Sun Yat-Sen University Cancer Center were obtained for the use of these clinical materials for research purposes. Clinical information on the samples is summarized in Table 1. The tumor specimens were recruited from paraffin blocks of 126 primary UCBs. Seventy-two cases of normal bladder mucosa from adjacent nonneoplastic bladder tissue of the same UCB patients, in paraffin blocks, were also obtained. None of the UCB patients included in this study had received preoperative radiation or chemotherapy. Tumor grade and stage were defined according to the criteria of the WHO and the 6th edition of the pTNM classification of the International Union Against Cancer (UICC, 2002).

### 2.2. Western Blotting Analysis

Equal amounts of whole cell and tissue lysates were resolved by SDS-polyacrylamide gel electrophoresis and electrotransferred onto a polyvinylidene difluoride membrane (Pall Corp., Port Washington, NY, USA). The tissues were then incubated with primary rabbit monoclonal antibodies against H3K27me3 (1 : 1000 dilution; Cell Signaling Technology, Beverly, MA, USA). The immunoreactive signals were detected with an enhanced chemiluminescence kit (Amersham Biosciences, Uppsala, Sweden). The procedures were conducted in accordance with the manufacturers' instructions. GAPDH antibody (1 : 2000 dilution; Sigma, St. Louis, MO, USA) was used as the loading control.

### 2.3. Construction of TMAs

The TMA was constructed according to a method described previously [15]. In brief, the paraffin-embedded tissue blocks and the corresponding histological hematoxylin-and-eosin- (H&E-) stained slides were overlaid for tissue TMA sampling. Duplicates of 0.6 mm diameter cylinders were punched from representative tumor areas of individual donor tissue blocks and reembedded into a recipient paraffin block at a defined position, using a tissue arraying instrument (Beecher Instruments, Silver Spring, MD, USA). In our constructed bladder tissue TMA, 3 cores of sample were selected from each primary UCB and normal bladder tissue. Multiple sections (5 *μ*m thick) were cut from the TMA block and mounted on microscope slides.

### 2.4. Immunohistochemistry (IHC)

IHC studies were performed using a standard streptavidin-biotin-peroxidase complex method. In brief, TMA sections were deparaffinized and rehydrated. Endogenous peroxidase activity was blocked with 0.3% hydrogen peroxide for 15 min. For antigen retrieval, tissue slides were boiled in 10 mM citrate buffer (pH 6.0) and microwave-treated for 10 min (H3K27me3) or in Tris (hydroxymethyl) aminomethane-EDTA buffer (pH 8.0) in a pressure cooker for 12 min (EZH2). Nonspecific binding was blocked with 10% normal rabbit serum for 20 min. The tissue slides were incubated with anti-H3K27me3 (1 : 50; Abcam, Cambridge, MA, USA) or anti-EZH2 (1 : 100; BD Transduction Laboratories, Franklin Lakes, NJ, USA) for 60 min at 37°C in a moist chamber. Subsequently, the slides were sequentially incubated with biotinylated rabbit antimouse immunoglobulin at a concentration of 1 : 100 for 30 min at 37°C and then reacted with a streptavidin-peroxidase conjugate for 30 min at 37°C and 3′-3′ diaminobenzidine as a chromogen substrate. The nucleus was counterstained using Meyer's hematoxylin. A negative control was obtained by replacing the primary antibody with a normal murine immunoglobulin. Known immunostaining positive slides were used as positive controls. 

To evaluate of the H3K27me3 and EZH2 IHC staining in different bladder tissues, the nuclear pattern of H3K27me3 and EZH2 in bladder tissues was recorded as positive expression. Nuclear immunoreactivity scores for H3K27me3 [16, 17] and EZH2 [8, 12, 18] proteins were calculated using previously validated respective scoring systems, respectively. For H3K27me3, the system used calculated the percentage of nuclei that stained positive for the H3K27me3 protein in multiples of 10. As the frequency of the percentage of positively stained cells in all tumor samples assessed for H3K27me3 was almost normally distributed and ranged from 0% to 100% and the median value was 50%, a 50% cut-off value was used to, categorize samples into high and low expression levels [16, 17]. For EZH2, the system scored nuclear EZH2 expression by recording the percentage of nuclei with EZH2 immunoreactivity and classified samples into two groups: low expression, where there was <50% positive cells; and high expression, when ≧50% of the cells showed nuclear immunoreactivity [8, 12, 18]. H3K27me3 and EZH2 expression levels were assessed by pathologists who were blinded to the clinicopathological data.

### 2.5. Statistical Analysis

All statistical analyses were carried out using the SPSS v. 13.0 statistical software packages (SPSS, Chicago, IL, USA). The relationship between H3K27me3 expression and clinicopathological characteristics was analyzed by the Chi-square test. Survival curves were plotted by the Kaplan-Meier method and compared using the log-rank test. Survival data were evaluated using univariate and multivariate Cox regression analyses. *P* values of less than 0.05 were considered to indicate statistical significance.

## 3. Results

### 3.1. Expression Patterns of H3K27me3 in UCB Cells and Tissues by Western Blotting

**Figure 1:**
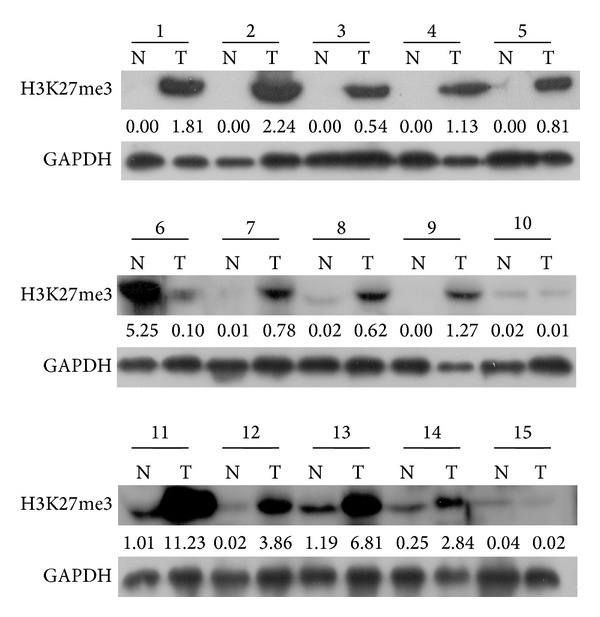
The expression of H3K27me3 protein in UCB tissues by Western bloting analysis. Upregulated expression of H3K27me3 was observed in 12/15 of UCB tissues compared to adjacent normal urothelial mucosal tissues. Expression levels were normalized with GAPDH.

To investigate the protein levels of H3K27me3 in UCB tissues, protein expression of H3K27me3 in 15 pairs of primary UCB and adjacent normal bladder specimens was analyzed using Western blotting. As shown in Figure 1, a total of 12 out of 15 (80.0%) UCB tissues samples had upregulated levels of H3K27me3 expression, compared with their adjacent normal bladder tissues. The results revealed that H3K27me3 was upregulated at the protein level, in clinical tissue samples of UCB.

### 3.2. The Expression Dynamics of H3K27me3 Examined by IHC in Bladder Tissue TMA

**Figure 2:**
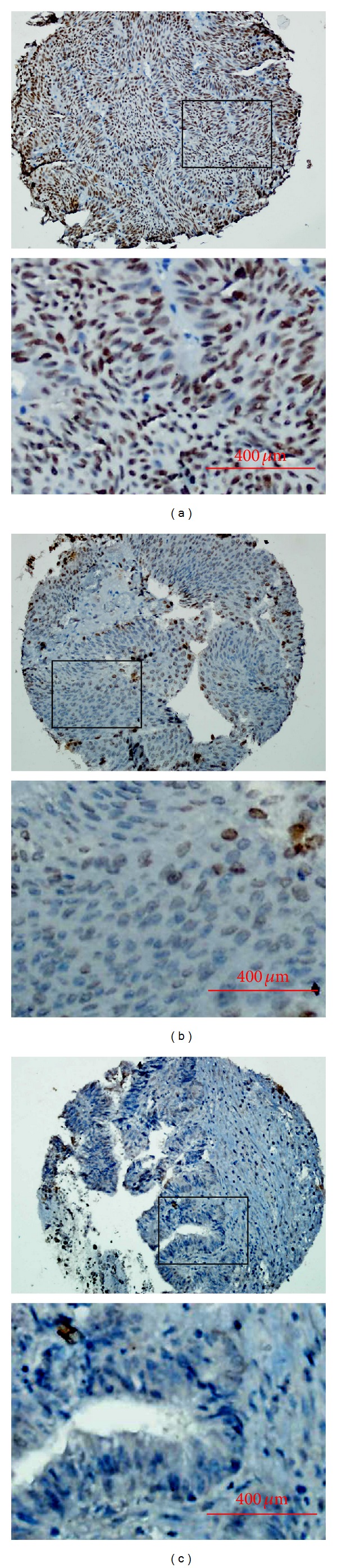
The expression of H3K27me3 in UCB and adjacent normal bladder tissues by IHC. (a) High expression of H3K27me3 was shown in a UCB case (case 25), in which more than 80% of carcinoma cells revealed positive staining of H3K27me3 protein in nuclei (100X). (b) Another UCB case (case 34) demonstrated low expression of H3K27me3, in which less than 30% of carcinoma cells showed positive staining of H3K27me3 protein in nuclei (100X). (c) Adjacent normal bladder urothelial mucosal tissue showed nearly negative expression of H3K27me3 protein (100X). The lower panels indicated the higher magnification (400X) from the area of the box in the upper panels.

In IHC study, immunoreactivity of H3K27m3 was observed primarily in the cell nuclei, though occasionally yellowish brown granules could also be seen in the cytoplasm (Figure 2). H3K27me3 expression could be evaluated informatively in 113/126 of UCB tissues and 61/72 of normal bladder tissues. The noninformative samples included unrepresentative samples, samples with too few tumor cells (<300 cells per case), and lost samples. For the noninformative TMA samples, IHC staining was replaced and performed by using whole tissue slides. By using the criteria (cutoff score) for H3K27me3 staining described before, high expression of H3K27me3 was examined in 59/126 (46.8%) of UCB and in 18/72 (25.0%) of normal urothelial bladder epithelial tissues (*P* = 0.002). The rates of high expression of H3K27me3 in UCBs with respect to several standard clinicopathologic features were detailed in Table 1. Correlation analysis demonstrated that high expression of H3K27me3 in UCBs was positively correlated with tumor multiplicity and N classification (*P* < 0.05, Table 1). There was no significant association between H3K27me3 expression and other clinicopathologic features, such as patient gender, age, tumor grade, and T classification (*P* > 0.05, Table 1). 

### 3.3. Relationship between Clinicopathologic Variables, H3K27me3 Expression and UCB Patient Survival: Univariate Survival Analysis

**Figure 3:**
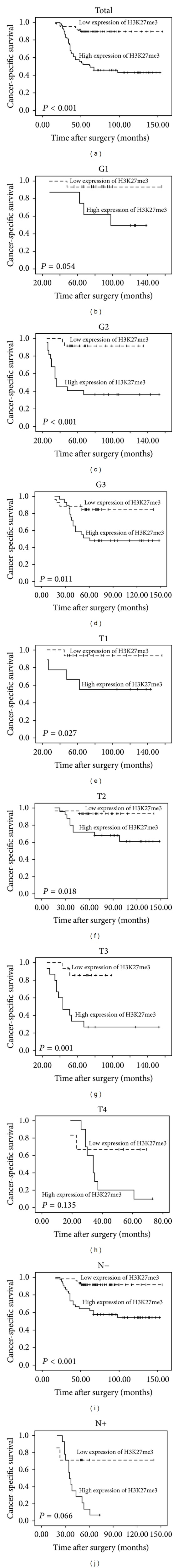
Kaplan-Meier survival analysis of H3K27me3 expression in patients with UCB (log-rank test). *Total, *probability of survival of all patients with UCB: low expression (*dashed line*), *n* = 67; high expression (*solid line*), *n* = 59. *G1, *probability of survival of G1 patients with UCB: low expression (*dashed line*), *n* = 16; high expression (*solid line*), *n* = 8. *G2, *probability of survival of G2 patients with UCB: low expression (*dashed line*), *n* = 24; high expression (*solid line*), *n* = 22. *G3, *probability of survival of G3 patients with UCB: low expression (*dashed line*), *n* = 27; high expression (*solid line*), *n* = 29. *pT1, *probability of survival of pT1 patients with UCB: low expression (*dashed line*), *n* = 16; high expression (*solid line*), *n* = 9. *pT2, *probability of survival of pT2 patients with UCB: low expression (*dashed line*), *n* = 30; high expression (*solid line*), *n* = 25. *pT3, *probability of survival of pT3 patients with UCB: low expression (*dashed line*), *n* = 15; high expression (*solid line*), *n* = 15. *pT4, *probability of survival of pT4 patients with UCB: low expression (*dashed line*), *n* = 6; high expression (*solid line*), *n* = 10. *pN*−, probability of survival of pN− patients with UCB: low expression (*dashed line*), *n* = 60; high expression (*solid line*), *n* = 45.* pN+, *probability of survival of pN+ patients with UCB: low expression (*dashed line*), *n* = 7; high expression (*solid line*), *n* = 14.

In univariate survival analyses, cumulative survival curves were calculated according to the Kaplan-Meier method. Differences in survival times were assessed with the log-rank test. Kaplan-Meier analysis showed a significant effect of certain clinical pathologic prognostic variables, such as tumor multiplicity (*P* = 0.026), tumor pT status (*P* < 0.001), and tumor pN status (*P* < 0.001) on patient survival (Table 2). Assessment of UCB patient survival also revealed that high expression of H3K27me3 was correlated significantly with poor cancer-specific survival (CSS, *P* < 0.001; Figure 3; Table 2). Additionally, survival analysis was done with regard to H3K27me3 expression in subsets of patients with different tumor histopathologic grades, pT and pN stages. The results showed that high expression of H3K27me3 was also a prognostic factor in UCB patients in grade 2 (*P* < 0.001) and grade 3 (*P* = 0.011), pT1 (*P* = 0.027), pT2 (*P* = 0.018), pT3 (*P* = 0.001), and pN− (*P* < 0.001; Figure 3). 

### 3.4. Independent Prognostic Factors of UCB: Multivariate Survival Analysis

As the variables observed to have prognostic influence by univariate analysis may be covariate, the expression of H3K27me3 and other clinicopathological features that were significant in the univariate analysis (tumor multiplicity, T classification, and N classification) were examined by a multivariate analysis (Table 3). We found that the high expression of H3K27me3 was an independent risk factor for adverse CSS (hazards ratio: 4.973; 95% confidence interval: 2.137–11.569; *P* < 0.001). Of the other variables, N classification also was found to be an independent prognostic predictor for CSS (Table 3).

### 3.5. Correlation between the Expression of H3K27me3 and EZH2 in UCB

**Figure 4:**
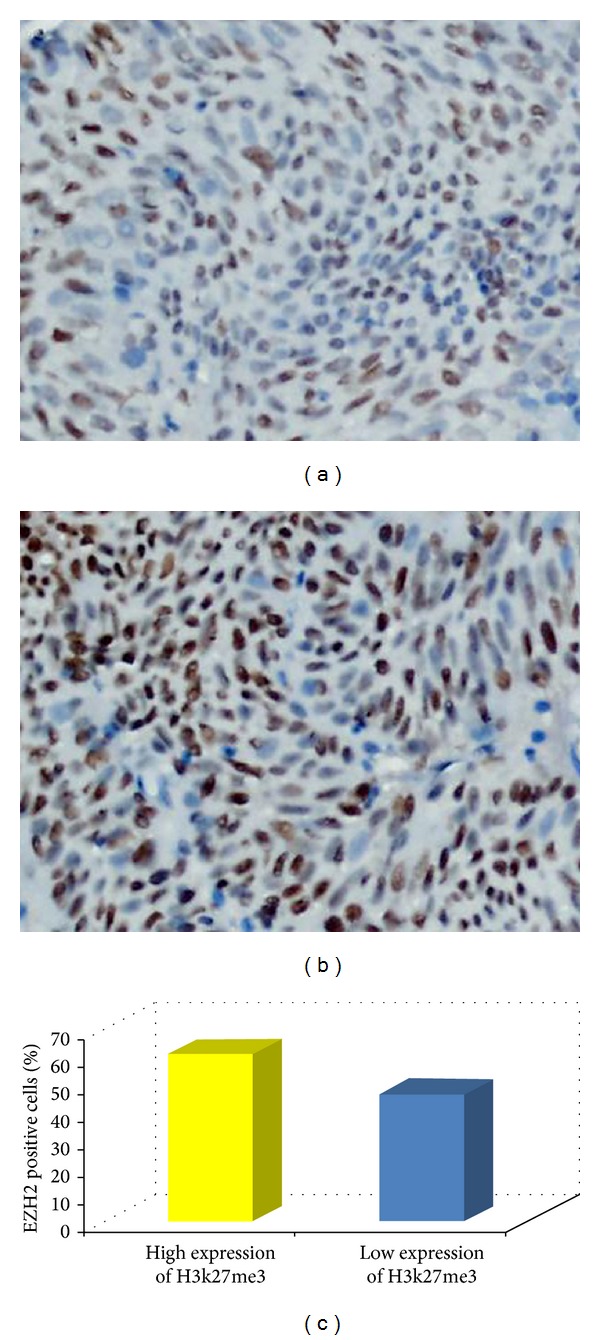
Correlation between expressions of H3K27me3 and EZH2 in UCB tissues. (a) High expression for EZH2 was observed in a UCB (case 86), in which more than 70% of tumor cells showed nuclear positive staining of EZH2 protein (400X). (b) Overexpression of H3K27me3 was examined in the same UCB case 86 (400X). (c) In 59 UCB cases with high expression of H3K27me3, an average of 61.0% of the UCB cells stained positive with EZH2, a percentage of cancer cells that was significantly larger than that (46.3%) in 67 UCBs with low expression of H3K27me3 (*P* = 0.003, independent sample *t* test).

For EZH2 staining, using the criteria described above, the high expression of EZH2 was observed in 67/126 (53.2%) of tissue samples of UCB, while the other 59 cases showed low EZH2 expression levels. Thus, we further evaluated the relationship between the expression of H3K27me3 and EZH2 in a cohort. The results showed a positive correlation between the expression levels of H3K27me3 and EZH2 (Figures 4(a) and 4(b)). For the 59 UCB cases with high H3K27me3 expression, 61.0% of carcinoma cells on average stained positive for EZH2 protein. This percentage was significantly higher than that of the 67 UCBs with low expression levels of H3K27me3 (46.3%; *P* = 0.003, independent sample *t* test; Figure 4(c)).

## 4. Discussion

Clinically, approximately 50% to 60% of patients diagnosed with muscle-invasive UCB will develop metastatic progression after local therapy with curative intent, resulting in approximately 12,000 deaths annually [2, 3]. Although current pTNM staging and histopathological grading systems have been established and are useful prognostic indicators for UCB after local therapy [19], these approaches are in the extent that they can provide information regarding patient prognosis and optimal treatment approaches. Patients with the same stage and/or grade of UCB treated with RC often display considerable variability in rates of disease recurrence and survival [20, 21]. Therefore, there is a need for new objective strategies that can effectively distinguish between patients with favorable and unfavorable prognoses. Individuals that are identified to have different prognoses by molecular biomarkers prior to surgery can have prolonged survival times with the addition of more effective adjuvant therapies [20]. Although UCB has been widely studied, the identification of specific genetic alterations associated with UCB tumorigenic processes and their clinical/prognostic significance remains substantially limited. Thus, further work is clearly needed to develop appropriate biomarkers.

Histone modifications are epigenetic mechanisms that play crucial roles in tumorigenesis [4]. One such modification, the trimethylation of H3K27, is mediated by proteins in the polycomb group family of genes. These were originally identified as genes that suppressed the development of extra sex combs in Drosophila [22]. It has been suggested that the maintenance of the H3K27me3 epigenetic mark during cell division is pivotal for normal embryogenesis and cell identity [23]. The methylation of H3K27 mediated by EZH2 has been implicated in the aggressive phenotype of cancer cells through the repression of a panel of tumor suppressor genes [24, 25]. The loss of function of these genes, in turn, locks stem/precursor cells into abnormal clonal expansion which begins the process of neoplastic initiation [26, 27]. This might provide a possible explanation for the observed association between H3K27me3 expression and advanced pN stage in UCB found in this current study. Moreover, an imbalance in H3K27 methylation owing to overexpression of EZH2 has been implicated in metastatic prostate and aggressive breast cancers [11, 28], in which a highly significant overlap between PRC2- and H3K27me3-occupied genes was observed [28]. To determine whether there was a potential correlation between the expression of EZH2 and H3K27me3 in UCB, we evaluated the expression status of these 2 proteins by IHC in the same cohort of cases. Our results demonstrated that the expression level of EZH2 in the high H3K27me3 expression group was significantly higher than that in the low H3K27me3 expression group, which supported the view that the upregulated expression of H3K27me3 in UCBs might be caused, at least in part, by the increased expression of EZH2. Moreover, H3K27 trimethylation has been shown to be correlated with the development and/or progression of different human cancers [5]. To date, however, the expression dynamics of H3K27me3 in UCB and its potential impact on UCB tumorigenesis and/or prognosis have not been elucidated. 

In the present study, we reported, for the first time, the clinical significance of H3K27me3 in UCB. This is also the first study that aimed to evaluate the possibility of using H3K27me3 as a clinically potential indicator for disease progression as well as a prognostic marker for patient survival in UCB. Our results, from Western blot analysis, indicated that the expression of endogenous H3K27me3 was upregulated in the majority of UCB tissue samples. Next, the expression dynamics of H3K27me3 was examined by IHC in a large cohort of UCB tissues samples taken from patients who underwent RC. These were analyzed using a bladder tissue TMA with complete follow-up data of each patient. Our results demonstrated that the frequency of high H3K27me3 expression in UCB tissues was significantly larger than that in nonneoplastic bladder epithelial tissues. These findings suggest that the upregulated expression of H3K27me3 may provide a selective advantage in UCB tumorigenic processes. Furthermore, we showed that the expression level of H3K27me3 protein significantly correlated with the clinical characteristics of UCB, including tumor multiplicity, N classification, and patient prognosis. These findings were similar to those of other studies [18, 29], in which the H3K27me3 protein was found to be frequently overexpressed in esophageal and hepatocellular carcinomas and positively correlated with tumor aggressiveness and/or advanced clinical stage. Taken together, these data suggest that the upregulation of H3K27me3 may facilitate the invasive/metastatic phenotypes of different types of human cancers, including UCB.

## 5. Conclusions

In this study, we reported for the first time that H3K27me3 expression was upregulated in clinical UCB tissues, and high expression of H3k27me3 was associated closely with a more malignant clinical feature and/or poor prognosis of UCB patients. Our results suggest that H3K27me3 overexpression might be useful as a prognostic factor for UCB patients. Apparently, a further understanding of the molecular mechanism by which H3K27me3 is involved in cancer cell initiation, proliferation, and/or transformation in human UCB would help in the discovery of novel targeted agents and might also lead to the development of new approaches for effective therapy of human UCB.

## Figures and Tables

**Table 1:** Association between the expression of H3K27me3 and clinicopathologic features in UCB.

Characteristic	Total cases	H3K27me3 expression (%)		
		Low expression	High expression	*P* value*
Age (years)				0.955
≤65**	68	36 (52.9)	32 (47.1)	
>65	58	31 (53.4)	27 (46.6)	
Gender				0.817
Male	114	61 (53.5)	53 (46.5)	
Female	12	6 (50.0)	6 (50.0)	
Tumor multiplicity				0.010
Unifocal	49	19 (38.8)	30 (61.2)	
Multifocal	77	48 (62.3)	29 (37.7)	
WHO grade				0.312
G1	24	16 (66.7)	8 (33.3)	
G2	46	24 (52.2)	22 (47.8)	
G3	56	27 (48.2)	29 (51.8)	
pT status				0.404
PT1	25	16 (64.0)	9 (36.0)	
PT2	55	30 (54.5)	25 (45.5)	
PT3	30	15 (50.0)	15 (50.0)	
PT4	16	6 (37.5)	10 (62.5)	
pN status				0.046
PN−	105	60 (57.1)	45 (42.9)	
PN+	21	7 (33.3)	14 (66.7)	

*Chi-square test; **median age; UCB: urothelial carcinoma of bladder.

**Table 2:** Univariate analysis of H3K27m3 expression and various clinicopathological parameters in 126 patients with UCB.

Characteristic	Total cases	RR (95% CI)	*P* value
Age, years			0.786
≤65*	68	1	
>65	58	1.090 (0.586–2.027)	
Gender			0.675
Male	114	1	
Female	12	0.777 (0.239–2.523)	
Tumor multiplicity			0.026
Unifocal	49	1	
Multifocal	77	2.025 (1.088–3.771)	
WHO grade			0.330
G1	24	1	
G2	46	2.069 (0.757–5.655)	
G3	56	2.016 (0.751–5.412)	
pT status			<0.001
PT1	25	1	
PT2	55	1.047 (0.363–3.017)	
PT3	30	2.561 (0.908–7.219)	
PT4	16	6.577 (2.250–19.227)	
pN status			<0.001
PN−	105	1	
PN+	21	4.957 (2.573–9.549)	
H3K27me3			<0.001
Low expression	67	1	
High expression	59	6.476 (2.857–14.676)	

*Median age; RR: relative risk; CI: confidence interval; UCB: urothelial carcinoma of bladder.

**Table 3:** Cox multivariate analyses of prognostic factors on survival.

Variable	Hazards ratio	95% CI	*P* value
Tumor multiplicity (unifocal versus multifocal)	1.352	0.704–2.595	0.365
pT status (PT1 versus PT2 versus PT3 versus PT4)	1.480	0.893–2.450	0.128
pN status (PN− versus PN+)	2.166	0.816–5.751	0.021
H3K27me3 (low versus high)	4.973	2.137–11.569	<0.001

CI: confidence interval.
